# The gut microbiome and metabolome of people living with HIV altered during treatment

**DOI:** 10.3389/fmicb.2026.1920281

**Published:** 2026-09-29

**Authors:** Xue Ding, Jiahe Li, Pengyu Qian, Jingyu Yue, Xiuxia Ma, Liran Xu

**Affiliations:** 1 The First Affiliated Hospital of Henan University of Chinese Medicine, Zhengzhou, China; 2 Tianjin University of Chinese Medicine, Tianjin, China; 3 Henan University of Chinese Medicine, Zhengzhou, China

**Keywords:** antiretroviral therapy, gut microbiome, HIV/AIDS, metabolome, Shen Ling Ai Cao pill

## Abstract

**Background:**

Despite effective viral suppression with antiretroviral therapy (ART), people living with HIV (PLWH) often experience persistent immune activation and incomplete immune reconstitution. Gut barrier disruption and gut microbial dysbiosis contribute to chronic inflammation in PLWH. Shen Ling Ai Cao pill (SLAC) may have immunomodulatory effects, however, changes in the gut microbiota and fecal metabolome during SLAC administration remain unclear. This study aimed to characterize changes in the gut microbiota and fecal metabolome during SLAC administration in PLWH.

**Methods:**

In this single-arm study, 46 PLWH on long-term ART received adjunctive SLAC for 6 months. Fecal samples were collected, and peripheral CD4+ and CD8+ T-cell counts and the CD4/CD8 ratio were measured at baseline and month 6. Gut microbiota were profiled by 16S rRNA gene sequencing, and untargeted metabolomics was performed to characterize fecal metabolic changes. Microbial diversity, differential taxa, functional pathways, differential metabolites, and KEGG enrichment were analyzed. Correlation analyses were conducted to explore associations among gut microbiota, metabolites, and T-cell subsets.

**Results:**

Among the 46 PLWH enrolled, 42 completed the 6-month follow-up and were included in the final analysis. From M0 to M6, median CD8+ T-cell counts increased from 448.5 to 565.0 cells/mm^3^ (*P* = 0.047), whereas median CD4+ T-cell counts increased non-significantly from 308.0 to 330.0 cells/mm^3^ (*P* = 0.118), with no change in the CD4/CD8 ratio (*P* = 0.89). Differences were also observed in gut microbial composition and fecal metabolomic profiles. Correlation analyses identified associations between gut microbial taxa and CD4+ and CD8+ T-cell counts. A total of 59 differential metabolites were identified, and enrichment analysis implicated lipid metabolism, bile acid metabolism, cholesterol metabolism, and mTOR signaling.

**Conclusions:**

In this single-arm longitudinal study of ART-treated PLWH, 6 months of adjunctive SLAC administration was associated with changes in fecal microbial composition, fecal metabolomic profiles, and CD8+ T cell counts, whereas CD4+ T cell counts showed a nonsignificant increasing trend. Controlled studies are needed to determine whether these changes are attributable to SLAC.

## Introduction

Human immunodeficiency virus/acquired immunodeficiency syndrome (HIV/AIDS) remains a significant global health burden (Correia et al., 2025). Antiretroviral therapy (ART) has transformed HIV infection into a manageable chronic disease by effectively suppressing viral replication and reducing AIDS related mortality (Deeks et al., 2013). However, ART does not achieve complete immune reconstitution in all PLWH. Many people living with HIV continue to experience persistent immune activation, chronic inflammation, and CD4^+^ T cell dysfunction which are associated with opportunistic infections, non AIDS related comorbidities, and impaired long term prognosis (Lederman et al., 2013; Fenwick et al., 2019; Manzardo et al., 2015; Li et al., 2025).

The gastrointestinal tract, harboring the largest pool of immune cells, is a primary site of immune depletion. In people living with HIV(PLWH), severe mucosal barrier damage and subsequent gut microbiota dysbiosis lead to microbial translocation, which chronically fuels systemic immune activation (Mazzuti et al., 2023; Zevin et al., 2016). Previous studies have described altered gut microbiota compositions in PLWH compared to healthy controls, characterized by a reduction in short chain fatty acids (SCFAs) producing bacteria (Dillon et al., 2017).

Although ART successfully suppresses viral replication, it cannot fully mitigate residual immune activation or gut mucosal dysbiosis. Moreover, long term ART is associated with adverse effects, drug resistance, and economic burden (Deeks et al., 2013; Bingham et al., 2021). Therefore, safe and effective adjunctive therapies are needed to support host immunity and gut health in PLWH. Traditional Chinese Medicine (TCM) formulas have shown potential in regulating immune responses, relieving symptoms, and improving CD4^+^ T cell levels (Ding et al., 2026, 2025). Among them, Shen Ling Ai Cao pill (SLAC) has attracted attention for its immunomodulatory and anti-inflammatory effects (Shao et al., 2022). SLAC may offer a benefit to PLWH by other mechanisms of action as well. Several clinical studies have reported varying effects on TCM symptoms, immune function, and microbiome composition when ART was supplemented with SLAC, although these studies varied widely in their methodologies and assessment metrics (Lan et al., 2025; Yang et al., 2024; Lan et al., 2024). However, the specific changes in gut microbial and metabolomic profiles during SLAC administration remain to be fully characterized.

In this single-arm longitudinal study, we integrated gut microbiome and untargeted fecal metabolome analyses to evaluate changes in gut microbiota and fecal profiles during 6 months of SLAC administration, as well as their potential associations with peripheral T-cell subsets in PLWH receiving stable ART.

## Materials and methods

### Patient information

A total of 46 PLWH were recruited from the First Affiliated Hospital of the Henan University of Chinese Medicine. All participants provided written informed consent, and this study was approved by the ethics committee of First Affiliated Hospital of the Henan University of Chinese Medicine (Approval No. 2025HL-439-01). The inclusion criteria were: participants aged 18–65 years diagnosed with HIV, receiving ART for more than 4 years, and with the TCM syndrome of spleen and kidney deficiency. All participants provided written informed consent prior to inclusion. Exclusion criteria included participants with severe cardiac, pulmonary, hepatic, or renal dysfunction, or who had participated in other clinical trials. The flowchart for this trial is shown in Figure 1.

**Figure 1:**
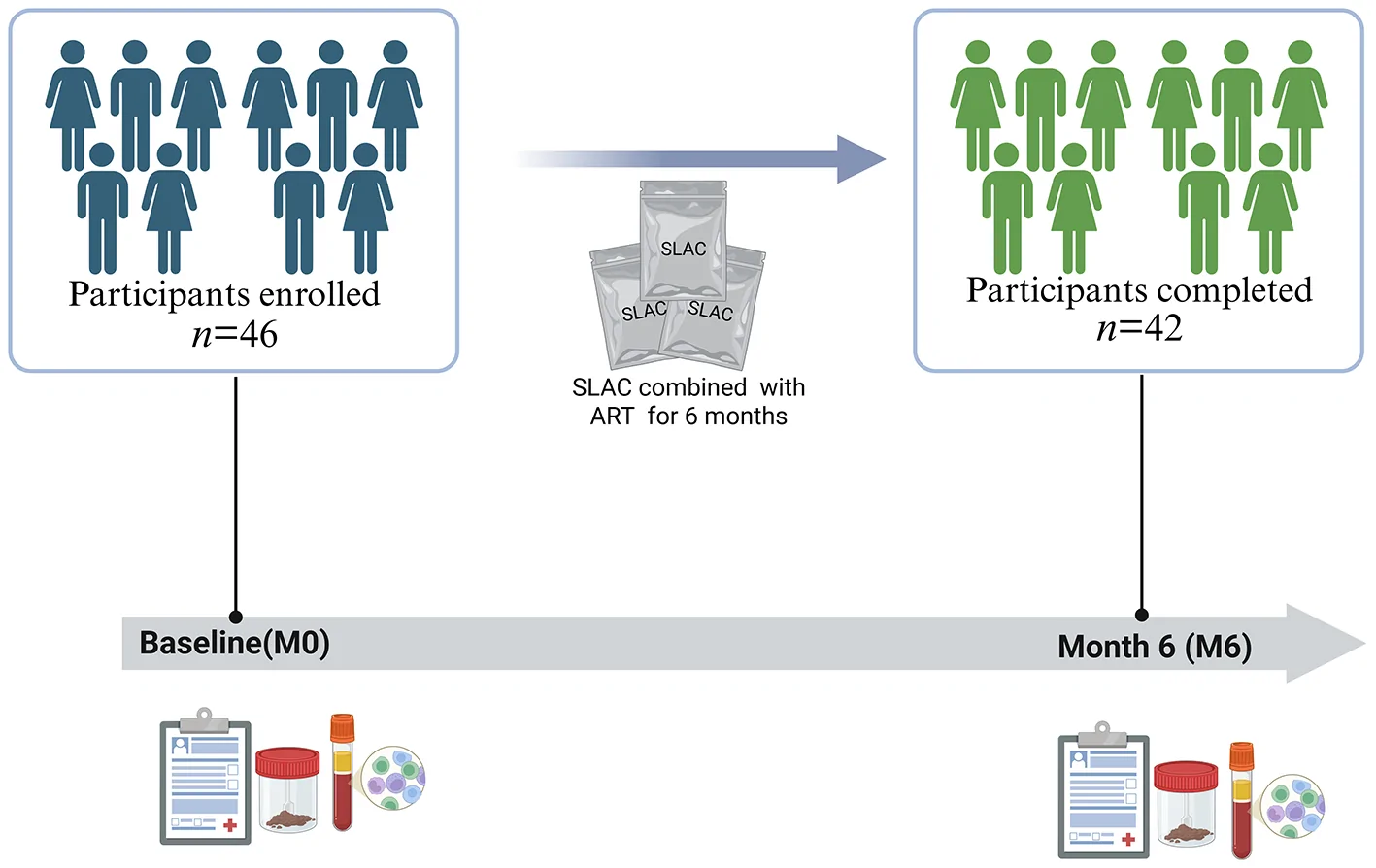
Study flow diagram. SLAC, ART, antiretroviral therapy; Shen Ling Ai Cao pill. Created in BioRender. Xue, D. (2026). https://BioRender.com/epvmn8o.

### Intervention

Samples were collected at baseline (M0) and after 6 months of treatment (M6). SLAC was manufactured by the Pharmaceutical Preparation Department of the Henan Academy of Chinese Medicine, with each sachet containing 5 g of the formulation. Participants received one 5g sachet orally three times daily, 1–2 h after meals, for 6 months. The formulation primarily contained processed sun-dried ginseng, *Astragalus* root, *Ganoderma*, and other herbal ingredients.

### Sample collection

Peripheral blood was collected at the same visit for routine clinical flow cytometric measurement of CD4+ and CD8+ T cell counts and calculation of the CD4/CD8 ratio. Participants collected morning fecal samples at the local hospital using the provided collection kits. Samples were labeled, immediately placed on dry ice without stabilizing buffer, stored at −80 °C, and transported to the laboratory on dry ice. They remained frozen at −80 °C until DNA extraction. Identical procedures were used at M0 and M6.

### DNA extraction

DNA was extracted from approximately 100 mg of fecal samples using the MagBeads FastDNA Kit for Soil according to the manufacturer's instructions. DNA concentration and quality were assessed using a NanoDrop spectrophotometer and agarose gel electrophoresis. The extracted DNA was stored at −20 °C until further analysis.

The V3–V4 regions of the bacterial 16S rRNA gene were amplified using primers 338F (5′-ACTCCTACGGGAGGCAGCA-3′) and 806R (5′-GGACTACHVGGGTWTCTAAT-3′) with 7-bp barcodes. PCR amplification was performed using FastPfu DNA Polymerase. Amplicons were purified, quantified, pooled in equimolar amounts, and sequenced on the Illumina NovaSeq 6000 platform using a paired end 2 × 250 bp strategy.

### Bioinformatics analysis

Following Illumina sequencing, raw reads were processed using QIIME2 (v2024.5). After demultiplexing and primer removal, quality filtering, denoising, paired end read merging, and chimera removal were performed using DADA2 to generate amplicon sequence variants (ASVs). ASVs were classified using Greengenes2 (version 2022.10), selected for its genome-informed taxonomy and compatibility with QIIME 2. After rarefaction to 28,000 sequences per sample, alpha diversity was evaluated using the Chao1, observed-species, Shannon, Simpson, and Pielou indices, with paired differences assessed using the Wilcoxon signed-rank test. Beta diversity was assessed using Bray-Curtis dissimilarities, visualized by PCoA, and tested using PERMANOVA with 999 permutations accounting for participant-level pairing.

Microbial biomarkers were identified using LEfSe with alpha < 0.05 and LDA score > 2.0. A genus-level Random Forest model was used to select the top 20 predictive features after excluding rare genera and log10 transforming the abundance data. Spearman correlation analysis was performed to assess associations between Z-score-normalized CD4/CD8 levels and the top 20 genera, with results visualized as heatmaps. Statistical significance was set at *P* < 0.05.

### Metabolomic profiling and bioinformatic analysis

Untargeted fecal metabolomics was performed using a Vanquish Flex UHPLC-Orbitrap Exploris 120 mass spectrometry system in positive and negative-ion modes, and data were processed using MS-DIAL (version 4.9.221218). Metabolites were annotated by matching retention time, accurate mass (< 10 ppm), and MS/MS spectra against in-house and public databases, followed by manual verification, achieving MSI confidence level 2 or higher. PLS-DA was performed, and univariate differences were assessed using Student's *t*-test. The resulting *P* values were adjusted for multiple comparisons using the Benjamini-Hochberg procedure. Metabolites with VIP > 1.0 and an FDR-adjusted *P* value < 0.05 were considered significantly differential. Pearson correlation analysis and hierarchical clustering were performed using log10-transformed metabolite abundances. A random forest model was used to rank and select core predictive metabolites. KEGG pathway enrichment *P* values were also adjusted using the Benjamini-Hochberg procedure.

### Statistical analysis

Demographic and clinical characteristics were analyzed using SPSS 26.0. Categorical variables are expressed as *n* (%), and continuous variables as mean ± SD or median (IQR). Paired comparisons between M0 and M6 were performed using the Wilcoxon signed-rank test. *P* < 0.05 was considered statistically significant.

## Results

### Participant characteristics and changes in T cell counts

A total of 46 PLWH were enrolled, and 42(91.3%) completed the 6 month follow-up. Overall, 86 fecal samples were collected and profiled, including 42 baseline samples and 42 follow-up samples. Pre-post analyses were restricted to participants with samples available at both time points (*n* = 42). The baseline demographic and clinical characteristics of all 42 enrolled participants are summarized in Table 1.

**Table 1:** Demographic data.

Characteristics	Total (*N* = 42)
Demographics	
Gender, male, *n* (%)	22(52.3%)
Age (years), median (range)	53.5 (45.0–65.0)
BMI, mean ± SD	22.75 ± 2.26
Comorbidities, *n* (%)	
Diabetes	10 (23.3%)
Hypertension	11 (25.6%)
Others	2(4.6%)
ART regimens, *n*(%)	
INSTI based	15 (34.9%)
PI based	16 (37.2%)
NNRTI based	10 (23.3%)
Others	2 2 (4.7%)
CD4 count (cells/mm^3^), median (IQR)	308.0 (248.0–424.0)
CD8 count (cells/mm^3^), median (IQR)	448.5 (380.0–601.3)

BMI, body mass index; ART, antiretroviral therapy; INSTI, integrase strand transfer inhibitor; PI, protease inhibitor; NNRTI, Non-nucleoside reverse transcriptase inhibitor.

From M0 to M6, the median CD4+ T-cell count increased from 308.0 cells/mm^3^ to 330.0 cells/mm^3^ (*P* = 0.118, Figure 2A). Meanwhile, CD8+ T-cell counts increased significantly from a median of 448.5 cells/mm^3^ at M0 to 565.0 cells/mm^3^ at M6 (*P* = 0.047, Figure 2B). No statistically significant change in the CD4/CD8 ratio was observed from M0 to M6 (*P* = 0.89). During the study, all participants remained virologically suppressed. No participants changed their ART regimen during the 6 month study period.

**Figure 2:**
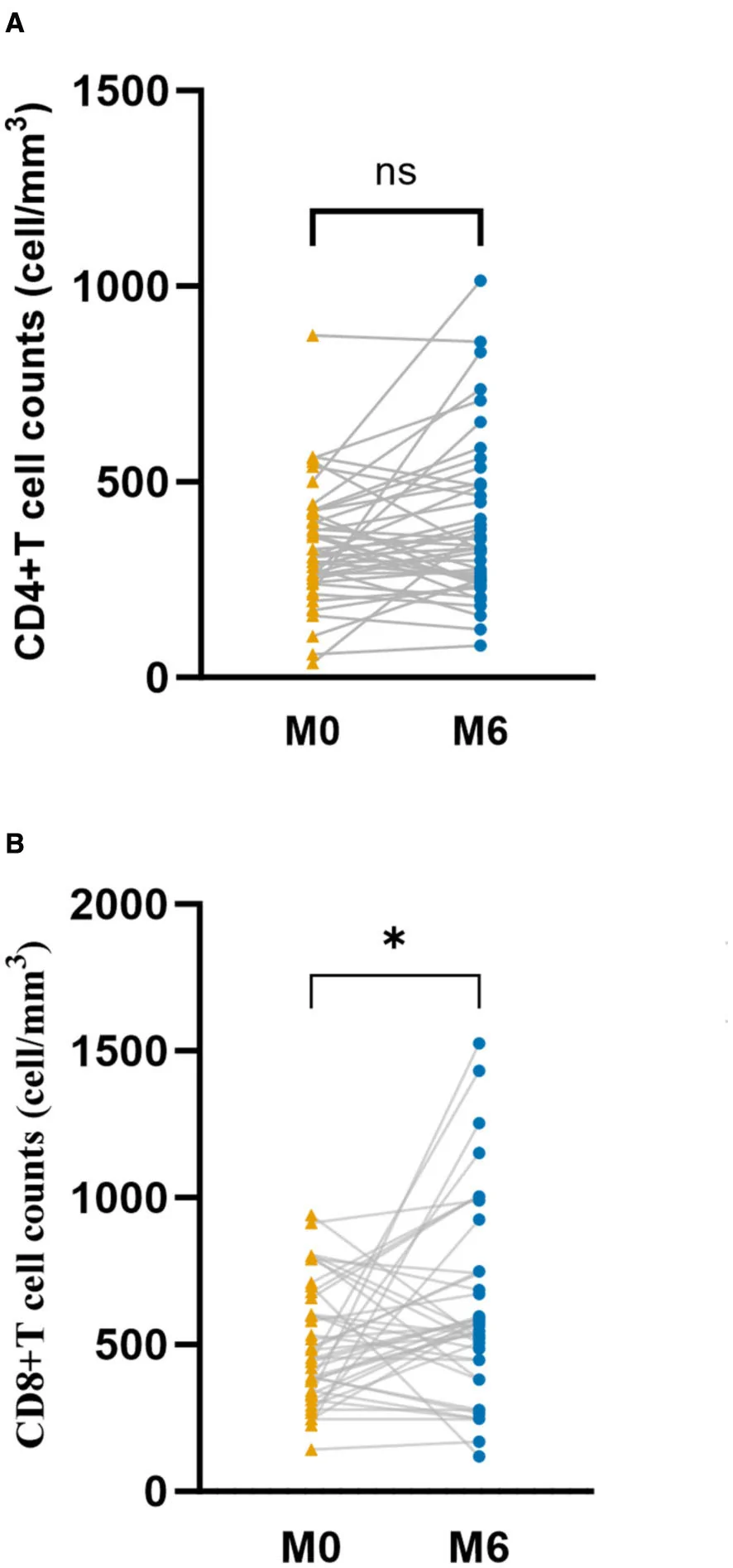
Changes in T cell counts between M0 and M6. **(A)** Changes in CD4^+^ T cell counts. **(B)** Changes in CD8^+^ T cell counts. *P < 0.05.

### Gut microbial composition

Gut microbial composition and structural shifts induced by SLAC using 16S rRNA gene sequencing. To evaluate the impact of SLAC on the gut microbiota, we undertook 16S rRNA gene sequencing on paired fecal samples before and after treatment. In total, 32,330 sequence variants were identified across all 86 fecal samples from 42 patients. After filtering for sequence variants present in at least 5% of the samples with a minimum average relative abundance of 0.01%, 567 sequence variants were retained for community analysis.

At the genus level, the fecal microbial communities at both M0 and M6 were dominated by *Prevotella, Faecalibacterium, Bifidobacterium, Phocaeicola_A, Escherichia, Blautia_A, Bacteroides_H*, and *Agathobacter* (Figure 3A). Descriptively, M6 samples showed higher relative abundances of *Prevotella, Faecalibacterium*, and *Escherichia* and lower relative abundances of *Bifidobacterium, Phocaeicola_A, Bacteroides_H*, and *Agathobacter* than M0 samples. At the ASV level, microbial richness was higher at M6 than at M0, as indicated by the Chao1 and observed-species indices (*P* = 0.011 and *P* = 0.015, respectively). In contrast, Pielou's evenness was lower at M6 (*P* = 0.032), whereas neither the Shannon nor Simpson diversity index differed significantly between the two time points (*P* = 0.39 and *P* = 0.076, respectively, Figure 3B). Bray-Curtis PCoA showed substantial overlap between M0 and M6, while PERMANOVA indicated a statistically significant difference in community composition (*R*^2^ = 0.0228, *P* = 0.001, Supplementary Figure S1).

**Figure 3:**
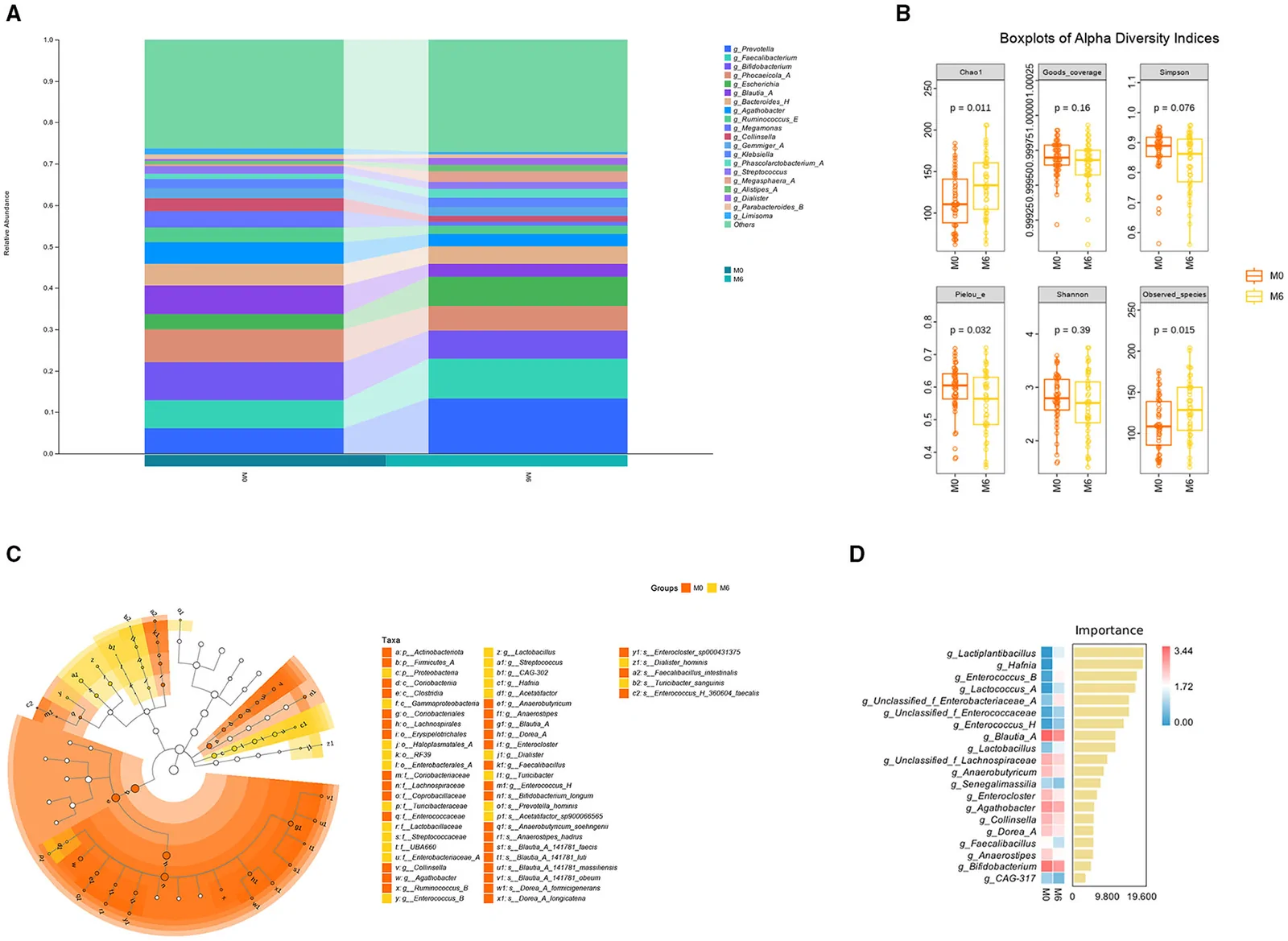
Gut microbial composition, diversity, and differential taxa. **(A)** Relative abundances of the 20 most abundant bacterial genera at M0 and M6. **(B)** Alpha diversity metrics of gut microbiota. **(C)** LEfSe cladogram showing differentially enriched bacterial taxa. **(D)** Random Forest analysis identifying key bacterial taxa.

### Taxonomic shifts and microbial biomarkers

LEfSe analysis was conducted to identify taxonomic features differentially enriched between M0 and M6 following SLAC treatment (Figure 3C). The cladogram revealed that the M6 group was characterized by significant enrichment of the phylum *Proteobacteria*, class *Gammaproteobacteria*, and family *Enterobacteriaceae_A*, whereas the M0 group was enriched in the phylum *Actinobacteriota*, class *Coriobacteriia*, and the genera *Blautia_A* and *Enterocloster*.

A Random Forest (RF) model was constructed to further identify key predictive biomarkers (Figure 3D). Among the top 20 influential taxa, *Lactiplantibacillus, Hafnia, Enterococcus_B*, and *Lactococcus_A* were identified as the major discriminative features. The relative abundance heatmap showed that *Hafnia* and *Enterococcus_B* were markedly reduced after treatment.

### Predicted functional shifts in the gut microbiota

Functional prediction based on 16S rRNA sequencing indicated that the gut microbiota at M0 and M6 differed in several predicted KEGG pathways, particularly metabolism-related pathways, including carbohydrate metabolism, amino acid metabolism, metabolism of cofactors and vitamins, and metabolism of terpenoids and polyketides (Figure 4A).

**Figure 4:**
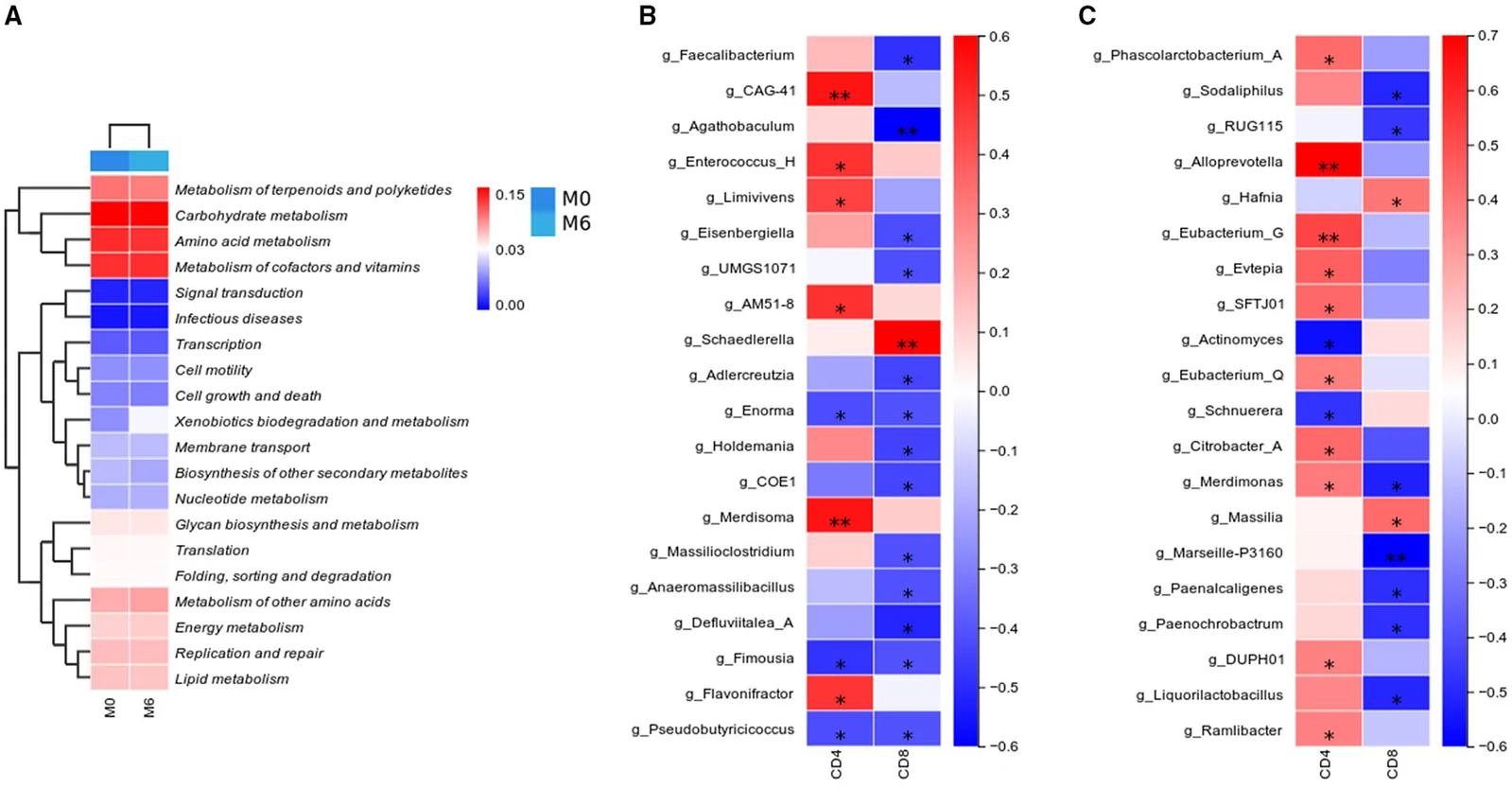
Predicted microbial functions and associations with immune markers. **(A)** Predicted functional profiles of the gut microbiota at M0 and M6. **(B)** Spearman correlations between bacterial taxa and CD4+ and CD8+ T cell counts at M0. **(C)** Spearman correlations between bacterial taxa and CD4+ and CD8+ T cell counts at M6. Colors represent Spearman correlation coefficients. *P < 0.05, **P < 0.01.

### Correlations between gut microbiota and immune markers

Spearman correlation analysis showed that the associations between gut microbiota and immune markers differed between M0 and M6. At M0, CD4 counts were mainly positively correlated with taxa such as *CAG-41, Merdimonas*, and *Flavonifractor*, and negatively correlated with *Enorma* and *Pseudobutyricicoccus*. CD8 counts exhibited predominantly negative correlations with several genera, including *Faecalibacterium, Agathobaculum, Eisenbergiella*, and *Pseudobutyricicoccus*, while showing a positive correlation with *Schaedlerella* (Figure 4B).

At M6, CD4+ T counts were positively associated with representative taxa such as *Alloprevotella, Eubacterium_G, Merdimonas*, and *Ramlibacter*, but negatively associated with *Actinomyces*. CD8+ T counts were positively associated with *Hafnia* and *Massilia*, whereas negative correlations were observed with taxa including *Sodaliphilus, RUG115, Merdimonas*, and *Liquorilactobacillus* (Figure 4C).

### Untargeted fecal metabolomics reveals alterations in fecal metabolic profiles

To assess the metabolic profile in the gut during TCM treatment, we undertook untargeted metabolomic profiling of fecal samples. Untargeted fecal metabolomics (POS mode) identified compounds. These predominantly comprised lipids and lipid-like molecules (28%), organic acids and derivatives (21%), organoheterocyclic compounds (20.3%), benzenoids (10.7%), phenylpropanoids and polyketides (6.9%), and organic oxygen compounds (4.7%) (Figure 5A). Key functional metabolites were detected, including the microbial tryptophan metabolite dl-3-indolelactic acid and pantothenate. Notably, pantothenate detection aligned with the microbial pantothenate and CoA biosynthesis pathway predicted by 16S rRNA analysis, linking microbial shifts to metabolic expression.

**Figure 5:**
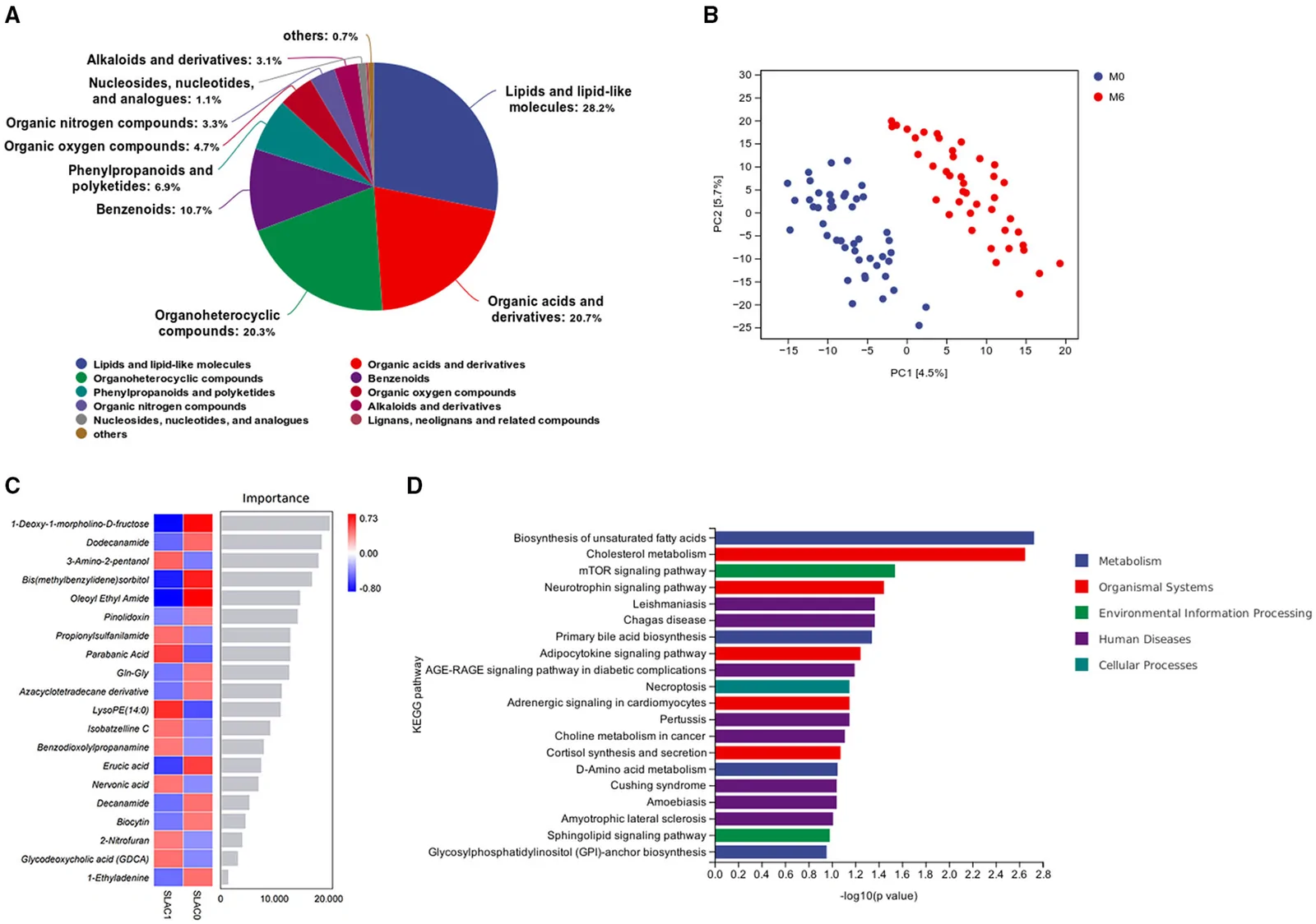
Fecal metabolomic profiles and differential metabolites at M0 and M6. **(A)** Chemical classification of metabolites identified in fecal samples. **(B)** PLS DA score plot acquired in positive and negative ionization modes (*R*^2^Y = 0.987; *Q*^2^ = 0.692). **(C)** Random forest analysis identifies key metabolic signatures. **(D)** KEGG pathway enrichment analysis of differential metabolites. PLS DA, partial least squares discriminant analysis; KEGG, Kyoto Encyclopedia of Genes and Genomes.

Supervised PLS-DA showed a clear separation between the M0 and M6 groups along the first latent component, which explained 4.5% of the total variation (Figure 5B). A total of 59 differential metabolites were identified between M6 and M0, including 22 increased and 37 decreased metabolites at M6 relative to M0 (Supplementary Figure S2). These findings suggest that SLAC treatment markedly remodels the gut metabolomic profile.

### Machine learning identifies key metabolic signatures

A random forest model was used to discriminate between M0 and M6 metabolic profiles. Metabolites were ranked based on their variable importance scores, identifying several top contributors to group separation, including 1-Deoxy-1-morpholino-D-fructose, Dodecanamide, 3-Amino-2-pentanol, Bis(methylbenzylidene)sorbitol, Parabanic Acid, and Gln-Gly (Figure 5C). Notably, 1-Deoxy-1-morpholino-D-fructose, Dodecanamide, Bis(methylbenzylidene)sorbitol, and Gln-Gly displayed reduced abundance at M6 compared with M0, whereas 3-Amino-2-pentanol and Parabanic Acid were enriched at M6. These metabolites may constitute a treatment-associated metabolic signature and provide potential biomarkers for monitoring SLAC-induced metabolic reprogramming.

### KEGG enrichment analysis reveals alterations in metabolic pathways

KEGG pathway enrichment analysis was performed to explore the potential functional implications of SLAC associated metabolic alterations (Figure 5D). The differential metabolites were mainly enriched in biosynthesis of unsaturated fatty acids and cholesterol metabolism [–log_10_(*P*) >2.5], both critical for immune cell membrane remodeling. Additionally, the mTOR signaling pathway a key rheostat for T cell activation and Primary bile acid biosynthesis were significantly modulated (*P* < 0.05). These results suggest that SLAC treatment is associated with coordinated changes in lipid homeostasis, bile acid metabolism, and mTOR related immune regulatory pathways, which may collectively contribute to SLAC induced metabolic.

## Discussion

This study integrated gut microbiome profiling based on 16S rRNA sequencing, untargeted metabolomics, and longitudinal changes in CD4+ and CD8+ T cell counts to characterize the biological alterations in PLWH receiving SLAC administration alongside stable ART. Following a 6 month SLAC intervention, PLWH exhibited a non-significant upward trend in CD4+ T cell counts and a statistically significant increase in CD8+ T cell counts. In Traditional Chinese Medicine theory, this dual pattern aligns with the principle of “Fuzheng” where the support of host defense is accompanied by the active mobilization of cellular immune responses (Li et al., 2024). Furthermore, these integrated multi-omic findings indicate that peripheral T-cell dynamics during SLAC administration are accompanied by shifts in gut microbial composition and fecal metabolic profiles, suggesting potential involvement of the gut-immune axis.

During the 6-month study period, adjunctive SLAC administration was associated with changes in the gut microbial community. Microbial richness increased at M6, whereas evenness decreased and the Shannon and Simpson indices remained unchanged. Although PERMANOVA detected a difference in community composition, the substantial PCoA overlap and effect size (*R*^2^ = 0.0228) indicated limited overall variation. Taxon-specific changes included lower relative abundances of *Hafnia* and *Enterococcus_B*, which may represent potentially favorable signals given their opportunistic potential and the reported enrichment of *Enterococcus* in PLWH with inadequate immunological responses to ART (Donato et al., 2008; Tian et al., 2025). However, the concurrent enrichment of Proteobacteria-related lineages and reduction of several putatively beneficial taxa indicate a heterogeneous pattern (Dinh et al., 2015; Dillon et al., 2014). Thus, SLAC administration was associated with selective taxonomic remodeling rather than a uniform change in microbial diversity. Predicted changes in several metabolic pathways and time-specific correlations between microbial taxa and T-cell counts suggest potential interactions among the gut microbiota, metabolism, and host immunity (Rocafort et al., 2019). However, these exploratory associations do not establish a role for the gut microbiota in immune recovery.

Untargeted fecal metabolomics was performed to explore metabolic features associated with SLAC administration. Fecal levels of dl-3-indolelactic acid (ILA), a microbial tryptophan metabolite, were elevated following 6 months of treatment. ILA acts as an endogenous ligand for the aryl hydrocarbon receptor (AhR), a pathway implicated in intraepithelial lymphocyte maintenance, IL-22 production, epithelial regeneration, and tight-junction maintenance (Ehrlich et al., 2020; Chen et al., 2025; Li et al., 2022). Pantothenate was also enriched, matching the PICRUSt2-predicted increase in microbial pantothenate and CoA biosynthesis. As pantothenate is essential for fatty acid synthesis and energy metabolism, its elevation may reflect metabolic processes relevant to mucosal health. These multi-omic results provide preliminary insights into potential associations among the gut microbiota, fecal metabolome, and host immune parameters during SLAC administration in PLWH.

Beyond individual metabolite alterations, pathway-level enrichment analysis provided further insight into the biological relevance of SLAC-associated metabolic changes. Enrichment analysis of differentially abundant fecal metabolites revealed significant involvement of mTOR signaling, unsaturated fatty acid biosynthesis, and cholesterol metabolism (Gabriel et al., 2021). These metabolic changes may be involved in T cell regulation because mTOR functions as a central nutrient and energy sensing pathway that regulates T cell activation, proliferation, differentiation, and survival (Buck et al., 2015). In PLWH, persistent inflammation, antigenic stimulation, and metabolic stress may impair T cell responsiveness. Therefore, changes in mTOR related metabolites may reflect a metabolic shift toward altered immune responsiveness. In addition, lipid metabolism is closely involved in T cell function, as cholesterol and unsaturated fatty acids influence membrane fluidity, lipid raft organization, TCR clustering, and immunological synapse formation (Hays et al., 2024). Thus, modulation of these pathways may provide a metabolic basis for the observed dynamics of CD4+ and CD8+ T cells.

There are several limitations in this study. First, the absence of a control group prevented us from distinguishing changes associated with SLAC from temporal variation, continued ART exposure, and unmeasured clinical, medication, dietary, or seasonal factors. Second, the follow up period was insufficient to assess the durability of immune changes. Third, 16S rRNA gene sequencing has limited taxonomic resolution and provides predicted rather than directly measured microbial functions, metagenomics and functional experiments are therefore needed for validation. Finally, these exploratory findings require mechanistic validation and confirmation in larger, diverse, randomized controlled studies.

In conclusion, this single-arm longitudinal exploratory study of ART-treated PLWH found that 6 months of SLAC administration was associated with changes in fecal microbial composition, fecal metabolomic profiles, and CD8+ T cell counts, whereas CD4+ T cell counts showed a nonsignificant increasing trend. These findings generate hypotheses regarding potential interactions among the gut microbiota, fecal metabolites, and immune parameters; however, controlled studies and mechanistic validation are required to determine whether these changes are attributable to SLAC and whether they contribute to immune recovery.

## Data Availability

The data analyzed in this study are available in the NCBI BioProject repository (https://www.ncbi.nlm.nih.gov/bioproject/PRJNA1527108) under accession number PRJNA1527108.
